# The History of a Case of Parturition Terminating Favourably without the Assistance of Art, Although the Uterus Formed a Herniâ without the Abdomen

**Published:** 1820-03

**Authors:** J. Saxthorp

**Affiliations:** Member of the Royal Medical Society of Copenhagen.


FOR THE LONDON MEDICAL AND PHYSICAL JOURNAL.

### *The History of a Case of Parturition terminating favourably without the assistance of Art, although the Uterus formed a Hernia without the Abdomen.* By J. Saxthorp, Member of the Royal Medical Society of Copenhagen.

ON the 20th of August, 1817, I was consulted by Mr. Sager, a provincial surgeon, respecting a woman who lived in the village of Hosterkiob, near Hirschholm, whom he believed to be affected with a hernia of the uterus in the state of pregnancy. He said this woman had been to consult him some days previously, complaining of a large tumor, which arose from the groin, covered the anterior and interior part of the right thigh, and descended as far as the knee. It was of a spherical form, covered by the common integuments in a state of great distension; it was elastic, not painful on being handled, and presented evident fluctuation. The patient, who had borne four children, declared that she felt in this tumor movements similar to those of a fetus; and Mr. Sager confirmed this statement, by asserting that he had discerned through the integuments of the tumor the form of a fetus. For two days past the woman had also experienced pains in the loins similar to those which precede labour. This account not being sufficient to enable me to form an opinion respecting the nature of the case, I requested that the woman might be immediately admitted into the Lying-in Hospital, where I might see her.

On the 24th of August she was brought in a cart, without suffering any other inconvenience during the journey than frequent desire to void her urine. The bulk of the tumor surprised me: it was as large and as heavy as the uterus in the seventh month of pregnancy; but I could not perceive the motions of a fetus. Fluctuation was, however, clearly evident in it; and I could discern here and there lines, or resistant and as it were tendinous bands, which I supposed to be what the surgeon had considered as the outlines of a fetus. On making an examination by the vagina, I found the orifice of the uterus at its upper part, similar to what it ordinarily is in women who have borne several children, but I could not discover the neck of it, nor recognize through the uterine orifice any body which appeared to be weighty. These observations were confirmed by Mr. Bang, junior, and by Mrs. Frost the chief midwife of the hospital. On examining with more care the origin of the tumor in the groin, I found that it commenced near Poupart's ligament, in such a way, however, that it did not pass under this ligament, but appeared rather to adhere to it. On placing my hand round its origin, above and below it, compressing as much as possible the integuments, it could not be observed that any part of the contents of the abdominal cavity passed into the tumor. The patient appeared to have been fatigued and emaciated by the severe labour in which she was habitually employed. It was with much difficulty I could get from her any explicit account of the antecedent circumstances of the case, from the weakness of her intellects. She had a fall on the ground, she said, in her infancy; after which she had a swelling appear in the right groin, for which she had employed no remedial measures, and which was productive of no inconvenience. Excepting that, she had always enjoyed good health. She had borne four children, and her labours had all been favourable. In her last pregnancy, four years before the present time, she had remarked this singular tumor in the groin, which was then only of the size of a turnip. But, in the month of April of the present year, it began to increase, and two months since it had acquired a more considerable size. In April, menstruation did not appear, which made the woman believe that she was pregnant, although she did not experience any other sign of that state, and she was in the forty-ninth year of her age. During the last eight days she had frequently suffered transient pains in the loins and belly, with some tenesmus, and frequent desire to void the urine.

On the 25th of August, the patient said she had passed the preceding night very unquietly, in consequence of the pains in the loins, which occurred after intervals. The ensuing night was more tranquil. The patient was then examined by Dr. Westberg, physician to the king of Sweden, and by several pupils at the hospital; but none of them could discern in the tumor motions similar to those of a fetus. On the 27th, Mrs. Frost told me that, having examined the tumor for half-an-hour on the preceding evening, she became convinced that there were within it motions precisely similar to those of a fetus bending and extending its limbs. I again examined it, but could not perceive those movements. The tumor had evidently increased in size during the last five days. As the existence of pregnancy was very doubtful, and the regulations of the hospital oppose the admission of any other than parturient women, I requested Mr. Thal to admit her into the Civil Hospital. The day after her entry there, Mr. Thal told me that he had discerned motions in the tumor which he had no doubt arose from the movements of a fetus.

On the 1st of October, Mr. Thal informed me that an inodorous, aqueous fluid, had flown from the vagina during the preceding night, without having been preceded, accompanied, or followed, by any pain. The sensation of tension which had been felt in the tumor was less painful, and its bulk was sensibly diminished. On the morning of the 2d of October, the patient was taken with severe pains in the tumor itself, which were intermittent, and accompanied by efforts like those of ordinary labour. The tumor presented contractions similar to those which the uterus effects in that state. The patient was again admitted into the Lying-in Hospital at six o'clock (in the morning). The midwife examined the state of the cervix of the uterus, and found that it was about an inch and a half in extent: she inserted her finger, and distinguished, exteriorly to, and below, the superior margin of the pelvis, the head of a fetus. An hour afterwards, having myself examined the patient, I also perceived the head, and besides that the navel-string. The pains continued with regularity and considerable force, without the labour, however, advancing in relative proportion to them. At ten o'clock I saw her, with Professors Fenger, Thal, and Bang: the dilatation of the os uteri was greater; the head of the fetus filled the superior aperture of the pelvis; the integuments of it began to become tumid, and a greater portion of the umbilical cord had fallen into the vagina. At one o'clock in the afternoon the pains were less severe, yet the head of the fetus was more advanced in the pelvis, and presented in a good position; but the dilatation of the cervix of the uterus had not much increased. At five o'clock the pains continued, the head advanced in the neck of the uterus, and the dilatation of this continued to proceed. At eight o'clock, the cervix of the uterus was completely effaced; the head of the uterus had penetrated into the pelvis, and the violence of the contraction drove it gradually towards the inferior aperture. I remained at the hospital, with Drs. Fenger, Thal, and Bang; and, by the powers of nature alone, the patient was, at nine o'clock, delivered of a dead female infant, eighteen inches in length, weighing five pounds and a half, and of which the head measured five inches and a half in diameter from the chin to the occiput. The nails, fingers, and toes, were soft and imperfectly developed; but, in other respects, the body and limbs were those of a well-constituted fetus.

After the delivery of the fetus, the tumor diminished in size: it did not descend so low as it had done, and could be easily raised upwards towards the abdomen. After having waited three-quarters of an hour in vain for the expulsion of the placenta, I introduced my hand into the uterus, and delivered it: no hemorrhage took place. The tumor, which I clearly recognized to be the uterus, preserved nearly the same bulk and form as after the expulsion of the fetus. I surrounded the belly by a large bandage, by the aid of which I raised up the tumor, to prevent its falling towards the thigh; and, after having given the woman a cordial, I had her put in bed. The consequences were fortunate: twenty days after delivery, the strength of the patient was completely re-established, and she returned home. A great part of the uterus remained without the abdomen, and formed a hernia, to which was joined a cyst filled with fluid, the presence of which was evident to the touch. It could now be more clearly perceived that the uterus had not passed through a natural opening, but between the fibres of the abdominal muscles.—*Nova Acta Regiæ Societatis Medicæ Hauniensis*, tom. i.

